# Lateralized Supraspinal Functional Connectivity Correlate with Pain and Motor Dysfunction in Rat Hemicontusion Cervical Spinal Cord Injury

**DOI:** 10.1089/neur.2022.0040

**Published:** 2022-10-03

**Authors:** Basavaraju G. Sanganahalli, Swathi Pavuluri, Jyothsna Chitturi, Peter Herman, Stella Elkabes, Robert Heary, Fahmeed Hyder, Sridhar S. Kannurpatti

**Affiliations:** ^1^ Department of Radiology, Rutgers Biomedical and Health Sciences–New Jersey Medical School, Newark, New Jersey, USA.; ^2^ Department of Radiology and Biomedical Imaging, Yale University School of Medicine, New Haven, Connecticut, USA.; ^3^ Department of Neurosurgery, Rutgers Biomedical and Health Sciences–New Jersey Medical School, Newark, New Jersey, USA.; ^4^ Hackensack Meridian School of Medicine, Mountainside Medical Center, Montclair, New Jersey, USA.

**Keywords:** fMRI, forepaw, functional connectivity, pain, spinal cord injury

## Abstract

Afferent nociceptive activity in the reorganizing spinal cord after SCI influences supraspinal regions to establish pain. Clinical evidence of poor motor functional recovery in SCI patients with pain, led us to hypothesize that sensory-motor integration transforms into sensory-motor interference to manifest pain. This was tested by investigating supraspinal changes in a rat model of hemicontusion cervical SCI. Animals displayed ipsilateral forelimb motor dysfunction and pain, which persisted at 6 weeks after SCI. Using resting state fMRI at 8 weeks after SCI, RSFC across 14 ROIs involved in nociception, indicated lateral differences with a relatively weaker right-right connectivity (deafferented-contralateral) compared to left-left (unaffected-ipsilateral). However, the sensory (S1) and motor (M1/M2) networks showed greater RSFC using right hemisphere ROI seeds when compared to left. Voxel seeds from the somatosensory forelimb (S1FL) and M1/M2 representations reproduced the SCI-induced sensory and motor RSFC enhancements observed using the ROI seeds. Larger local connectivity occurred in the right sensory and motor networks amidst a decreasing overall local connectivity. This maladaptive reorganization of the right (deafferented) hemisphere localized the sensory component of pain emerging from the ipsilateral forepaw. A significant expansion of the sensory and motor network s overlap occurred globally after SCI when compared to sham, supporting the hypothesis that sensory and motor interference manifests pain. Voxel-seed based analysis revealed greater sensory and motor network overlap in the left hemisphere when compared to the right. This left predominance of the overlap suggested relatively larger pain processing in the unaffected hemisphere, when compared to the deafferented side.

## Introduction

Human cervical spinal cord injury (SCI) is the most prevalent, affecting clinical functions and often accompanied by neuropathic pain.^1^ Rehabilitation after cervical SCI is often focused on regaining finer hand functions to improve quality of life.^2,3^ Given that hand functions like reaching and grasping are largely controlled by the brain rather than spinal circuitry, supraspinal reorganization studies in pre-clinical animal models of cervical SCI are critical for improved clinical rehabilitation strategies.^4,5^ Supraspinal reorganization of neural circuits start within hours after an SCI to modify sensory and motor representations in both humans and animal models.^5,6^ Patients with spinal, brainstem, thalamic, lenticular, or cortical lesions, and presenting with unilateral allodynia, show lesser contralateral activity (because of deafferentation) during innocuous stimulation of the pain territory. However, ipsilateral expansion of activity occurs in the motor and pre-motor areas (M1; supplementary motor area), spatial attention areas (posterior parietal cortices), and areas linking attention and motor control (midanterior cingulate cortex), which may manifest pain processing.^7^ Amidst a somatotopic reorganization of the supraspinal motor network after an SCI in mostly pain-free patients,^8^ such reorganization is not only impeded in SCI patients with neuropathic pain,^9^ but also accompanied by poor motor recovery.^10^

The scarcity of supraspinal studies in animal models of cervical SCI is a barrier for deeper understanding of post-injury reorganization patterns and pre-clinical advancement of novel pain treatments. Further, non-invasive translatable imaging approaches in live animals are also necessary to bridge the pre-clinical gap with larger clinical SCI investigations in humans.^6^ Therefore, the aim of this study was to determine the supraspinal reorganization in the pre-clinical rat model of hemicontusion cervical SCI, exhibiting lateralized pain behavior. The functional magnetic resonance imaging (fMRI) approach, as applied in humans to decipher the nexus between pain and poor clinical motor functional recovery after SCI,^10^ was used.

The rat hemicontusion cervical SCI at the C2–C5 level leads to a permanent forelimb debilitation despite recovery of hindlimb activity^11,12^ and translates well to human cervical SCI.^13^ Hemicontusion rat cervical SCI with negligible sparing of ipsilateral spinal cord tissue at the epicenter results in both fore- and hindlimb motor debilitations accompanied by at- and below-level pain.^14^ However, a relatively larger sparing of ipsilateral spinal fibers results in only at-level pain.^15^ Our hemicontusion cervical SCI model in rats, which utilized a precise 150-kDyne force with a 2.5-mm impactor tip at the C4–C5 level and lateralized to one hemisphere, resulted in ipsilateral SCI lesions with a relatively larger sparing of gray and white matter tissue.^16^ We hypothesized that hemicontusion SCI, which leads to lateralized forepaw motor deficit and pain as observed in our model, will reflect a lateralized supraspinal functional reorganization. In our previous study, young adult rats (2 months of age) underwent sham or cervical SCI and were behaviorally monitored for sensorimotor responses from 1 to 6 weeks after SCI and pain at 5 and 6 weeks after SCI followed by resting-state functional fMRI at 8 weeks after the SCI. Using task fMRI, electrical stimulation of the debilitated ipsilateral forepaw led to reduced functional response across the affected (contralateral) S1_FL_ cortex, indicating deafferentation.^17^ From the resting-state fMRI data obtained in the same animal groups during those experiments, the current study tested our hypothesis by characterizing spontaneous brain activity within and across hemispheres.

Region of interest (ROI)-level seed-^17^ and voxel-level seed-based^18^ resting-state functional connectivity (RSFC) analysis of resting-state fMRI measures, which are standard data-driven methods to map the human brain connectome, was performed. ROI based RSFC was determined using 14 regions, many of them implicated in nociception^17^ and which showed significant changes in functional connectivity density (FCD) in these animals in our earlier study.^17^ Voxel-level seed-based RSFC, using the somatosensory forelimb cortical (S1_FL_) and primary/secondary motor cortical (M1/M2) representations, was determined with seed voxels originating bilaterally or separately from the left or right hemispheres.

## Methods

Female Sprague-Dawley rats 1.5 months of age were procured from Taconic Biosciences (Rensselaer, NY) and randomly assigned to sham (*n* = 7) or SCI (*n* = 11) groups. Baseline sensorimotor behavioral measures were obtained 1 week before sham or SCI procedures performed at 2 months. Food (standard chow) and water were provided *ad libitum* during the experiments, and all animal procedures were approved by the Rutgers University and Yale University institutional animal care and use committees in accordance with National Institutes of Health guidelines.

### Surgery and spinal cord injury induction

After anesthesia with ketamine/xylazine (100 mg/10 mg/kg, intraperitoneal [i.p.]), a midline incision was made from the base of the skull to the scapula, exposing the left-sided dorsal elements of the C2–C5 vertebrae. Muscles were retracted with four homemade retractors^19^ and pulled apart using a rongeur for an optimal surgical field for the hemilaminectomy. After laminectomy of the C5 column, animals were mounted on the Infinite Horizon Impactor device (Precision Systems and Instrumentation LLC, Lexington, KY) for the sham or SCI procedures. An impactor tip of 2.5 mm in diameter, along with a 150-kDyne force setting, was utilized to perform a moderate mechanical-impact contusion injury as described in our previous study.^16^ Sham animals underwent identical hemilaminectomies, including the muscle dissections without the impact injury. Post-injury, animals were provided 2 mL of saline i.p. to prevent dehydration and buprenorphine SR (0.03 mg/kg, subcutaneous) for analgesia immediately after surgery and monitored twice a day during the first 48 h after surgery and daily thereafter until 6 weeks. Post-operative care, including follow-up behavioral testing, was performed at Rutgers Biomedical and Health Sciences. At the end of 6 weeks, after completion of the behavioral experiments, animals were transferred to Yale University. After a quarantine of 2 weeks to ascertain the health of the animals after transfer, MRI experiments were performed 8 weeks after SCI.

### Magnetic resonance imaging

Magnetic resonance imaging (MRI) and fMRI were performed using a Bruker 9.4T spectrometer (Bruker Corp., Billerica, MA) and an ellipsoidal surface coil (5 × 3 cm). Animals were initially anesthetized with 1.5% isoflurane, and a PE 50 tubing was placed into the i.p. cavity for dexmedetomidine (sedative) infusion. Thereafter, animals were maintained under complete anesthesia with 0.3% isoflurane and 250 μg/kg/h i.p, of dexmedetomidine. Body temperature was maintained at 36°C–37°C using a heating pad and monitored using an MRI-compatible rectal probe throughout the MRI experiments. Before the fMRI measure, anatomical MRIs were acquired over 12 contiguous coronal brain slices (thickness = 1 mm), covering the parenchyma between the olfactory bulb and cerebellum. Anatomical reference images (repetition time [TR]/echo time [TE] = 4000/30 ms, 2 averages) were acquired in a 128 × 96 matrix, using a T2-weighted turbo rapid acquisition with relaxation enhancement sequence, providing an in-plane resolution of 250 μm × 250 μm for a field of view of 3.2 cm. Additionally, a fast three-dimensional anatomical scan (TR/TE = 50/5.6 ms, flip angle = 20 degrees, 2 averages) was acquired with an isotropic resolution of 250 μm, for image registration purposes. fMRI was performed across the 12 anatomical coronal slice positions (covering the brain) using a gradient-recalled echo planar imaging (GE-EPI) with a TR/TE = 1000/15 ms, 64 × 48 matrix, and 500 × 500 μm in-plane resolution). The GE-EPI sequence produced an image contrast sensitive to blood-oxygenation-level–dependent (BOLD) changes.^20^ Three resting-state fMRI scans, each lasting 512 sec (512 acquisitions), was obtained across each animal. Anatomical MRI scans of the cervical spinal cord and task-related fMRI scans were also acquired in the same animals as part of a different recently published study.^16^ The order of task and resting-state fMRI scans was randomized across animal subjects.

### Statistical analysis

The first sham rat's brain image was non-linearly registered to the brain anatomical images of all other subjects using the BioImage Suite^21^ with 50 iterations, normalized mutual information, and otherwise default. The average image of all subjects' registered images was obtained and subsequently non-linearly registered to the SIGMA rat brain template (https://www.nitrc.org/projects/sigma_template) to obtain the “study template” from which standard anatomical ROIs were extracted from regional masks (Supplementary Fig. S1). Brain anatomical images from each animal were subsequently registered to the study template brain using a non-linear registration (50 iterations, normalized mutual information, and otherwise default). Functional images were coregistered between rats after slice-timing correction followed by motion correction (to the middle volume) using SPM12 (The FIL Methods group, 2015; www.fil.ion.ucl.ac.uk/spm/software/spm12). Subsequently, the corresponding animal's non-linear transformation to the study template brain was applied to all functional images using BioImage Suite software. To reduce image noise and facilitate interanimal comparisons, data were blurred in every slice using a Gaussian filter (σ = 2 voxels/0.250 mm, size = 8 voxels/1 mm). The resting-state BOLD fMRI time-series data were linearly detrended to remove temporal signal drifts and band-pass filtered between 0.005 and 0.100 Hz before further analysis.

All seed ROI-based RSFC determinations were performed using custom programs in MATLAB (The MathWorks, Inc., Natick, MA). Fourteen anatomical ROIs (Supplementary Fig. S1) showing significant FCD changes in SCI when compared to sham, determined by our earlier study in the current animal groups,^17^ were considered. BOLD-fMRI time series across voxels in each of the 14 ROIs were averaged to obtain the mean BOLD time series of each respective seed ROI.^22^ Subsequently, the mean BOLD time series across each seed ROI from the left or right hemisphere were cross-correlated with the mean BOLD time series from every other ROI within the brain. RSFCs between seed regions were determined as a color-coded correlation matrix of z-scores after a Fisher z-transform of the correlation coefficient values.

All seed voxel-based RSFC determinations were performed using AFNI. Six random voxels from the S1_FL_ or M1/M2 anatomical regions respectively were selected from each hemisphere. Individual RSFC maps were obtained after cross-correlating the BOLD-fMRI time series from each seed voxel with all other voxels within the brain. This process was repeated across the six random seed voxels from each brain hemisphere, yielding six individual RSFC maps. Six seed voxels per hemisphere × 3 experimental runs × 7 animals yielded 126 individual RSFC maps for sham and 6 seed voxels per hemisphere × 3 experimental runs × 11 animals, yielding 198 individual RSFC maps for SCI. Average left, right, or both-hemisphere RSFCs were determined after converting the correlation coefficients in the individual RSFC maps to z-values using a Fisher z-transform. Average RSFC maps for both hemisphere seed voxels were obtained from 252 individual RSFC maps from the 7 sham animals and 396 individual RSFC maps from the 11 SCI animals. Seed voxel-based RSFC maps were color-coded based on the z-values and overlaid on the atlas-transformed (study template) T1-weighted MRI. Significant differences in voxel-level RSFC between SCI and sham conditions were statistically determined using a two-tailed *t*-test with a threshold of *p* < 0.05 and corrected for multiple comparisons by a family-wise error control using a contiguous cluster size of 30 voxels at least sharing one side.

## Results

A permanent ipsilateral forelimb motor deficit from 1 to 6 weeks after SCI with complete motor recovery across other remaining limbs has been reported in sham and SCI animal groups used for the current study.^16^ A thermal hypersensitivity test (Hargreaves), performed in a subset of 5 sham and 5 SCI animals, showed decreased limb withdrawal latency across the ipsilesional (left) forelimb after SCI when compared to sham at 5 and 6 weeks, respectively, post-SCI. Limb withdrawal latency, however, did not significantly differ between sham and SCI across all other limbs. These pain behavioral results have been recently reported.^17^ Significant changes in FCD occurred after SCI across 14 anatomical ROIs in the current group of SCI animals^17^ and were considered as seed regions for our RSFC investigation. Differing from FCD, which identifies nodes of spontaneous neuronal activity,^23^ RSFC indicates the strength of spontaneous neuronal activity and directionality of connections across regions.^17^

**FIG. 1.**
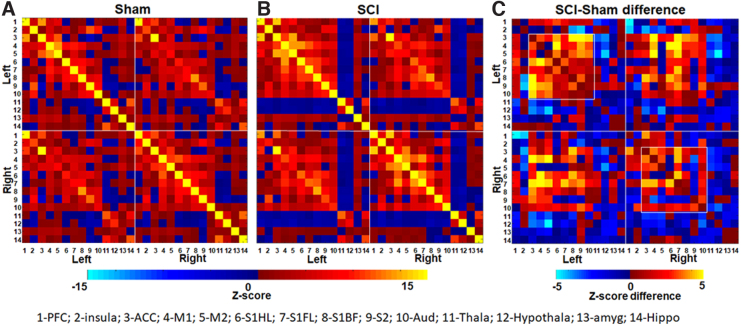
RSFC correlation matrix from sham and SCI animals. Fourteen anatomical regions of interest (ROIs) formed the seed region from either the left or right brain hemisphere. (**A**) RSFC correlation matrix from the sham group (*n* = 7). (**B**) RSFC correlation matrix from the SCI group (*n* = 14) and (**C**) RSFC difference between the SCI and sham groups. Colors represent z-score values or z-score differences. RSFC, resting-state functional connectivity; SCI, spinal cord injury.

Whole-brain and hemisphere-specific measures were obtained. Figure 1A,B shows the RSFC correlation matrix from the sham and SCI animal groups respectively across the 14 seed ROIs derived from either the left (ipsilateral) or right (contralateral) brain hemispheres. RSFC increased across ROIs 1–10 (1-PFC-Pre-frontal cortex, 2-Ins-Insula, 3-Acc-Anterior cingulate cortex, 4-M1-Primary motor cortex, 5-M2-Secondary motor cortex, 6-S1_FL_-Primary sensory forelimb cortex, 7- S1_HL_-Primary sensory hindlimb cortex, 8-S1_BF_-Primary sensory barrel field cortex, 9-S2-Secondary sensory cortex, 10-Aud-Auditory cortex) and ROI-14 (14-Hip-Hippocampus) in SCI when compared to sham. RSFC decreased across ROIs 11–13 (11-Th-Thalamus, HT-12-Hypothalamus, 13-Ag-Amygdala) in SCI when compared to sham **(**Fig. 1C). Intrahemispheric or local functional connectivity differences were observed across several cortical regions with increased RSFC within the left hemisphere (left-left connections) after SCI when compared to the right (right-right connections; Fig. 1C; square boxes**)**.

SCI also increased the interhemispheric RSFC (between the left and right) in select regions, such as the anterior cingulate cortex (ACC; ROI-3), pre-frontal cortex (PFC; ROI-1), insula (ROI-2), and the somatosensory (ROIs: 6–9) and auditory cortices (ROI-10) when compared to sham (Fig. 1C). Interhemispheric RSFC, however, decreased in other select regions, such as the thalamus (ROI-11), hypothalamus (ROI-12), amygdala (ROI-13), and hippocampus (ROI-14), in SCI when compared to sham (Fig. 1C).

**FIG. 2.**
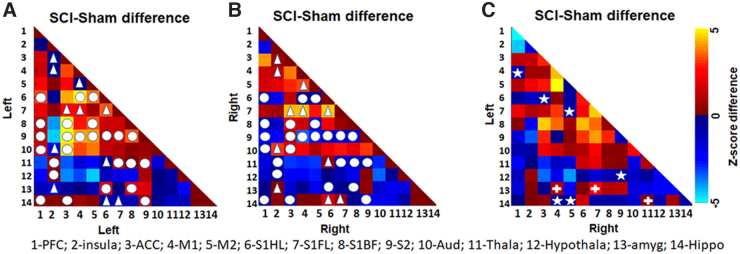
Inter- and intrahemisphere connectivity changes brought about by SCI. (**A**) Left-left RSFC difference between SCI and sham (**B**). Right-right RSFC difference between SCI and sham and (**C**) left-right RSFC difference between SCI and sham. Colors represent z-score differences. Circle represents intrahemispheric RSFC, which increased between two regions in the left hemisphere after SCI, showing a decrease between the same regions in the right hemisphere after SCI. Triangle represents intrahemispheric RSFC, which decreased between two regions in the left hemisphere after SCI, showing an increase between the same regions in the right hemisphere after SCI. Star represents the RSFC, which decreased between two regions across the hemispheres after SCI, but showed an increase within hemispheres after SCI. Plus (“+”) represents the RSFC, which increased between two regions across the hemisphere after SCI, but showed a decrease within hemispheres after SCI. RSFC, resting-state functional connectivity; SCI, spinal cord injury.

Intrahemispheric RSFCs, which increased between two regions locally in the left hemisphere after SCI, showed a decrease between the corresponding regions in the right hemisphere and were in a majority (Fig. 2, circles). Intrahemispheric RSFCs, which decreased between two regions locally in the left hemisphere after SCI, increased between the corresponding regions in the right hemisphere after SCI and were in a minority (Fig. 2, triangles). Interhemispheric global connectivity was also modulated after SCI, with RSFC decreasing between regions across hemispheres after SCI, which otherwise had increased RSFC between themselves within the hemisphere (Fig. 2, stars). On the other hand, RSFC increased between two regions across the hemisphere after SCI, which otherwise had decreased RSFC between themselves within the hemisphere (Fig. 2, plus signs). In summary, whereas SCI-induced intrahemispheric RSFC changes were clearly distinct across hemispheres, select interhemispheric connection strengths were swapped compared to their intrahemispheric directional changes, specifically the sensory cortical, motor cortical, hypothalamus, amygdala, and hippocampal regions (Fig. 2, stars and plus signs).

**FIG. 3.**
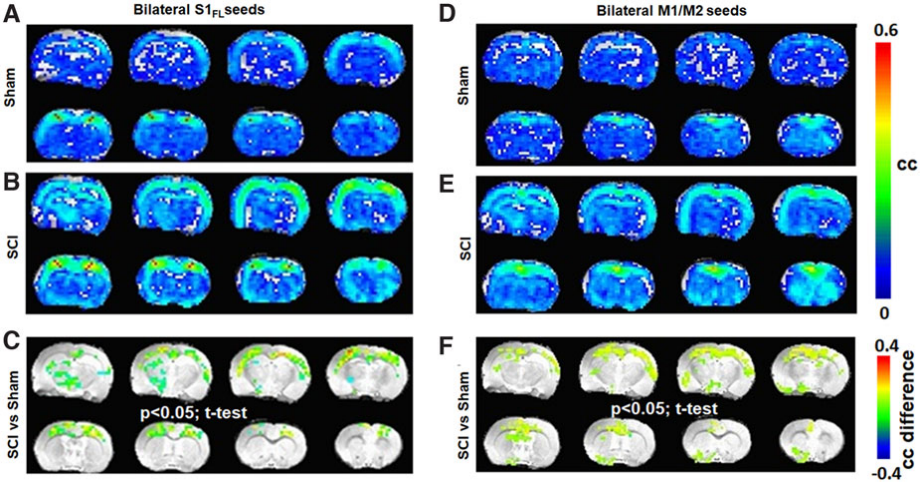
Average RSFC obtained using seed voxels from both hemispheres across sham (*n* = 7) and SCI groups (*n* = 11). (**A–C**) S1_FL_ representation and (**D–F**) M1 representation. Color bar represents the correlation coefficient (cc). Significant difference map of correlation coefficient values (SCI > sham; color bar representing the difference in cc). Activated voxels were statistically determined using a two-tailed *t*-test with an activation threshold of *p* < 0.05 and corrected for multiple comparisons by a family-wise error control using a cluster size of 30 voxels. Average RSFC maps represent 12 seed voxels × 3 experimental runs × 7 animals = 252 for sham and 12 seed voxels × 3 experimental runs × 11 animals = 396 for SCI. Image orientation is in the neurological convention (image left/right is subject left/right). RSFC, resting-state functional connectivity; SCI, spinal cord injury.

To determine brain functional consequences of the ipsilateral forelimb showing behavioral motor deficits and pain, the S1_FL_ RSFC network was specifically determined from the resting-state fMRI data. The average RSFC network was generated using 12 random seed voxels originating bilaterally from the S1_FL_ ROIs (six from each brain hemisphere) and cross-correlated with all other voxels from the brain across each animal. The S1_FL_ RSFC network from seed voxels originating bilaterally differed between sham and SCI, with a relatively stronger and larger spatial extent across SCI (Fig. 3A,B). Significant differences between SCI and sham (Fig. 3C; SCI vs. sham, two-tailed *t*-test, *p* < 0.05) were observed across the somatosensory cortex (mostly symmetric) and across the thalamus (mostly asymmetric). RSFC of the motor (M1/M2) network determined from 12 seed voxels originating bilaterally from the M1 and M2 ROIs showed RSFC increases across SCI (Fig. 3E) when compared to sham (Fig. 3D). Significant differences between SCI and sham (Fig. 3F; SCI vs. sham, two-tailed *t*-test, *p* < 0.05) were observed across the M1, M2, S1, and S2 cortices and across the hippocampus, thalamus, and septum.

**FIG. 4.**
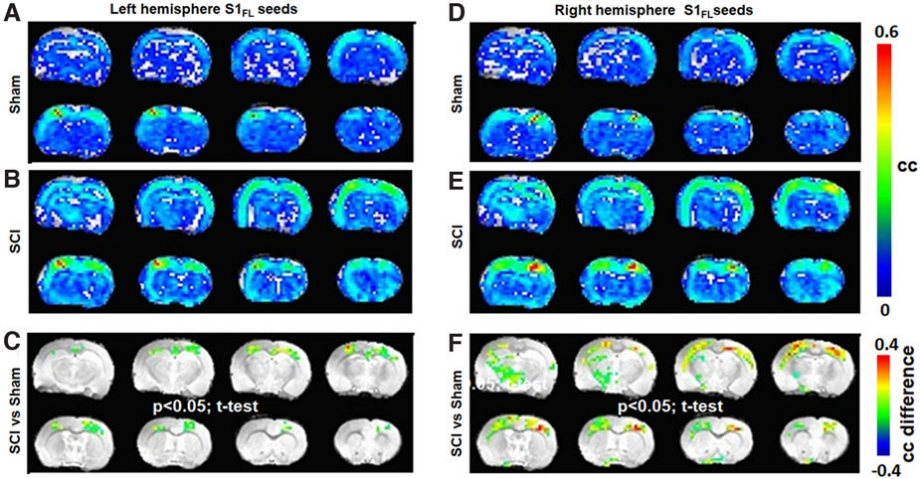
Average RSFC obtained using S1_FL_ seed voxels across sham (*n* = 7) and SCI groups (*n* = 11) from the (**A–C**) left hemisphere and (**D–F**) right hemisphere. Color bar represents the correlation coefficient (cc). Significant difference map of correlation coefficient values (SCI > sham; color bar representing the difference in cc). Activated voxels were statistically determined using a two-tailed *t*-test with an activation threshold of *p* < 0.05 and corrected for multiple comparisons by a family-wise error control using a cluster size of 30 voxels. Average RSFC maps represent 6 S1_FL_ seed voxels × 3 experimental runs × 7 animals = 126 for sham and 6 S1_FL_ seed voxels × 3 experimental runs × 11 animals = 198 for SCI. Image orientation is in the neurological convention. RSFC, resting-state functional connectivity; SCI, spinal cord injury.

From the average S1_FL_ RSFC networks obtained separately from the left or right hemisphere seed voxels, the SCI group showed consistently stronger S1_FL_ RSFC (Fig. 4B,E) when compared to sham (Fig. 4A,D). Whereas the left S1_FL_ seed RSFC network significantly differed between SCI and sham only across the cortex and displayed corticocortical symmetry (Fig. 4C), the right S1_FL_ seed RSFC network significantly differed between SCI and sham across both corticocortical and -thalamic connections (Fig. 4F). Further, the SCI versus sham differences were also asymmetric across brain hemispheres, with larger spatial extents over the left somatosensory cortex and left thalamic areas (Fig. 4C). The SCI versus sham difference was relatively less across the left S1_FL_ seed RSFC network when compared to the right (Fig. 4C), which had a relatively stronger and spatially larger extension over to the left thalamic areas (Fig. 4F).

**FIG. 5.**
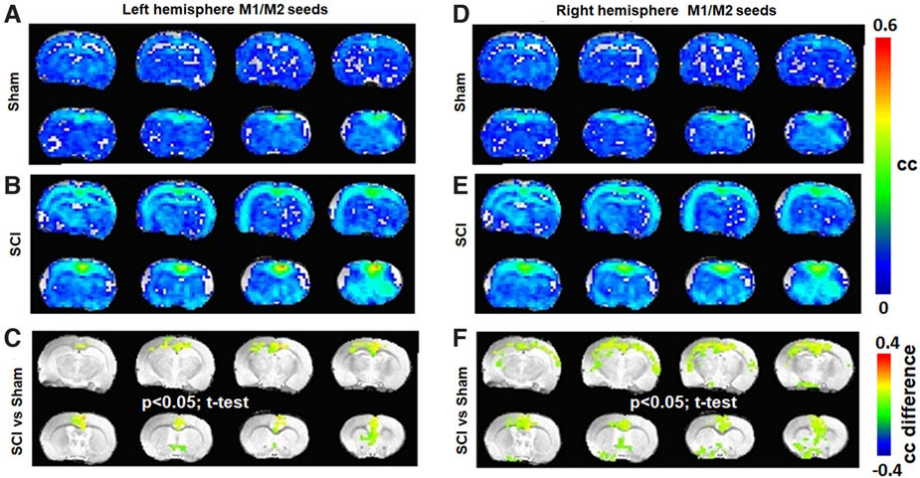
Average RSFC obtained using M1 seed voxels across sham (*n* = 7) and SCI groups (*n* = 11) from the (**A–C**) left hemisphere and (**D–F**) right hemisphere. Color bar represents the correlation coefficient (cc). Significant difference map of correlation coefficient values (SCI > sham; color bar representing the difference in cc). Activated voxels were statistically determined using a two-tailed *t*-test with an activation threshold of *p* < 0.05 and corrected for multiple comparisons by a family-wise error control using a cluster size of 30 voxels. Average RSFC maps represent 6 M1 seed voxels × 3 experimental runs × 7 animals = 126 for sham and 6 M1 seed voxels × 3 experimental runs × 11 animals = 198 for SCI. Image orientation is in the neurological convention. RSFC, resting-state functional connectivity; SCI, spinal cord injury.

From the motor RSFC networks obtained from only the left or right hemisphere seed voxels, the SCI group showed significantly stronger and larger connectivity (Fig. 5B,E) when compared to sham (Fig. 5A,D). Whereas the left motor seed RSFC network differed more strongly between SCI and sham and was localized to the M1 and M2 regions only (Fig. 5C), the right motor seed RSFC network differed between SCI and sham across the M1, M2, and S1 regions with relatively lesser RSFC strength, but larger spatial extents (Fig. 5F).

**FIG. 6.**
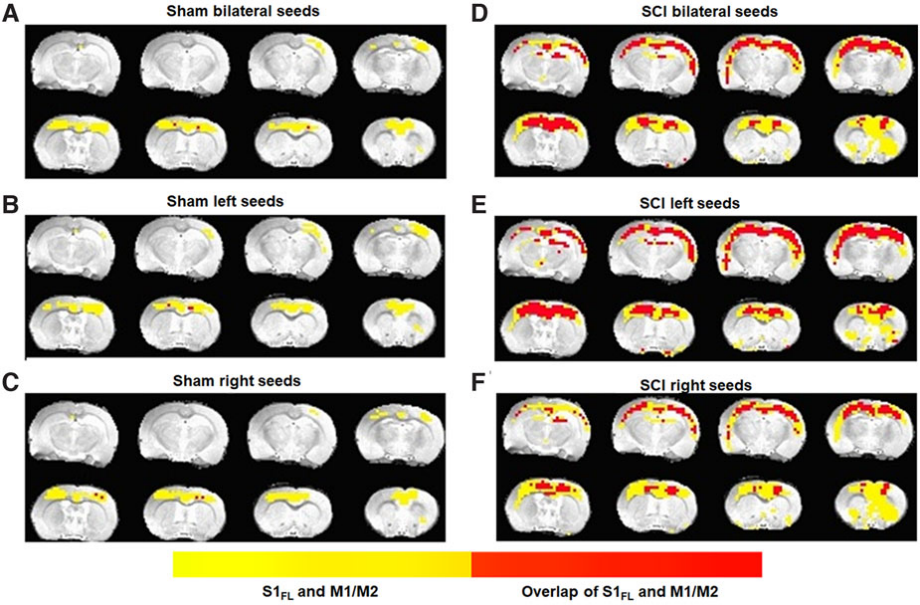
Average spatial extent of the S1_FL_ and M1/M2 resting-state networks (yellow) and their overlap (red) across sham (*n* = 7) and SCI (*n* = 11) groups. The z-transformed RSFC maps were averaged across animals in each group and subsequently inverse z-transformed to obtain the average correlation coefficient value map. A threshold of cc = 0.15 was used to generate the sensory and motor RSFC networks respectively. Image orientation is in the neurological convention. RSFC, resting-state functional connectivity; SCI, spinal cord injury.

Average spatial extents of the sensory and motor networks and their spatial overlaps were estimated across the sham and SCI groups. Sensory and motor network RSFCs were found to be spatially distinct with a small overlap across sham (Fig. 6A–C), whereas SCI showed a significantly increased overlap (Fig. 6D–F). A lateralization of sensory-motor overlap after SCI was also observed with a relatively larger sensory-motor overlap within the left seeds RSFC network (Fig. 6E), when compared to the right seeds RSFC network across the SCI group (Fig. 6F).

## Discussion

Hemicontusion cervical SCI in the rat demonstrated asymmetric RSFC changes across supraspinal sensory and motor networks. Stronger local connectivity within the right hemisphere sensory and motor networks in the background of an overall lower right-right connectivity, reflected deafferentation and maladaptive activity localizing the ipsilateral forelimb pain. An enhanced sensory-motor network overlap was observed globally after SCI when compared to sham, supporting the hypothesis that sensory and motor interference manifests pain.

Brain-wide RSFC alterations occurred after SCI, encompassing several nociceptive regions and consistent with earlier reported parenchymal structural and functional changes associated with chronic pain in SCI patients and animal models.^24–27^ Frontal brain areas, such as the PFC, insula,^28,29^ somatosensory cortex (S1 and S2), ACC,^30^ thalamus, periaqueductal gray, and amygdala, have been implicated in central nociception and correspond well in both human and animal models.^31,32^ Whereas the somatosensory cortices encode sensory features, such as location, intensity, and duration of pain,^33–35^ the ACC, insula, and amygdala (limbic system areas) encode the emotional (affective) component.^32,36,37^ Our seed ROI-based RSFC changes with increased or decreased connectivity across specific brain regions revealed a systematic pattern of supraspinal nociceptive activity in the SCI animals (Fig. 1). Distinct changes within the nociceptive ROIs across each hemisphere (Fig. 2) indicated that pain processing was supraspinally lateralized.

Sensory deafferentation is a hallmark of cortical reorganization when sensory deprivation occurs because of impaired sensory inputs,^38^ including pathological conditions such as SCI.^39^ Sensory deafferentation was ascertained by the lower S1_FL_ activation in response to electrical stimulation of the ipsilateral (left) forepaw in our earlier study in these SCI animals.^17^ The current results corroborated the presence of SCI-induced deafferentation from hemispheric asymmetry in local connectivity with relatively greater left-left connections than right-right across most cortical ROIs (Fig. 1C, correlated regions within the squares). Amidst the relatively greater left-left local connectivity attributable to deafferentation of the right hemisphere (Fig. 2A,B, circles), sparsely increased right-right connectivity was observed in specific regions when compared to corresponding regions on the left hemisphere (Fig. 2A,B, triangles). These sparsely increased connectivity induced by SCI in the deafferented hemisphere was observed between the insular cortex and ACC, M1, auditory cortex, and amygdala. Additionally, S1_FL_ connectivity in the deafferented hemisphere also increased with ACC, M1, S1_HL_, and hippocampus, along with increased connectivity between M1 and M2 (Fig. 2A,B, triangles). In summary, specific local RSFC increases in the deafferented (right) hemisphere after SCI (Fig. 2A,B, triangles), within the background of an overall decrease in right-right connectivity, indicated maladaptive nociceptive activity localizing pain in the ipsilateral forelimb. Interhemispheric connectivity decreased between S1_FL_ and M2 after SCI, which otherwise showed within-hemisphere increases between themselves across both hemispheres (Fig. 2, stars), signifying a reduced bilateral sensorimotor coordination. A similar trend was observed between M1 and PFC, hippocampus, and M1/M2 (Fig. 2, stars). Conversely, interhemispheric connectivity increased between amygdala and M1, amygdala and S1_FL_, and between hippocampus and thalamus, which otherwise showed within-hemisphere decreases between themselves across both hemispheres (Fig. 2, plus), indicated changes within the spinolimbic system after SCI.

Despite the apparent deafferentation in the current SCI animals, S1_FL_ seeds from the right (deafferented) hemisphere showed relatively larger RSFC intensity and spatial extent (Fig. 4F), compared to the left (unaffected; Fig. 4C), indicating a clear lateralized reorganization of somatosensory activity. A similar expansion of RSFC spatial extent was observed using the M1/M2 voxel seeds from the right (deafferented) cortex after SCI (Fig. 5F), when compared to the left (unaffected; Fig. 5C), pointing to a pain-related modulation of the motor cortical network. Functional neuroimaging studies in SCI patients have demonstrated relationships between the intensity of deafferentation pain and degree of deafferentation-related reorganization of the primary somatosensory cortex, which is reversible by attenuating pain.^40^ Similarly, somatotopic reorganization, required for regaining a certain level of motor activity after SCI, is not only impeded by pain,^9^ but also accompanied by poorer motor recovery.^10^ From these current pre-clinical results (Figs. 4 and 5), we posit that sensory and motor processing may interfere at the supraspinal level and persist within the widespread global background of nociceptive activity across several brain regions after SCI.^17^ The sensorimotor interference manifesting pain may impede somatomotor plasticity and subsequently reflect as poor motor improvements in pain-positive SCI patients.^10^

To confirm whether such an interference of sensory and motor circuits existed in the current animal model, we estimated the sensory and motor RSFC overlaps and observed that the respective networks were spatially distinct with small overlaps across the sham group (Fig. 6A–C). However, SCI showed significantly increased overlaps (Fig. 6D), in addition to lateralization (Fig. 6E,F). Whereas a small overlap of the sensory and motor networks was expected in the sham group attributable to normal sensorimotor integration activity of higher cortical areas, the expansion of this overlap after SCI confirmed a possible pain-related sensorimotor interference. Additionally, the left-voxel seed network was relatively more dominant than the right-voxel seed network in contributing to the expanded sensory-motor overlap after SCI (Fig. 6E,F). The greater contribution of left seeds to the sensory-motor overlap was completely opposite, when compared to its lesser contribution to RSFC strength and spatial extents across both S1_FL_ (Fig. 4C) and M1/M2 after SCI (Fig. 5C). In a similar manner, the right seeds contributed to relatively larger RSFC strength and spatial extents (Figs. 4F and 5F) and lesser to sensory-motor overlap (Fig. 6E). Further studies are needed to explain the opposite nature of RSFC strength and sensory/motor network overlap. Animal models of motor rehabilitation after SCI with and without pain treatments may also help establish the sensory network overlap as a pain biomarker and whether overlap minimization may improve motor recovery after SCI.

Sensory and motor networks from the left hemisphere seed overlapped to a greater extent than the right. The relatively greater left laterality of the overlap, taken together with the higher local connectivity of the sensory and motor networks of the left (unaffected) hemisphere (Fig.2), indicated that the unaffected ipsilateral hemisphere may contribute more to the processing of pain afferents when compared to the deafferented contralateral hemisphere. Compensatory neuroplasticity has been shown to originate through interhemispheric spinal fiber collateralization above and below the hemicontusion injury in SCI animal models.^11,41,42^ Hence, the right (uninjured) hemisphere of the spinal cord, in the current model, is highly likely to act as a relay for the afferent and efferent signals, bypassing the partially atrophied ipsilateral cord region to connect to the left (unaffected) hemisphere.

Invasive and non-invasive stimulation of the central nervous system, explored for neuropathic pain prevention, have been extremely encouraging.^43–46^ However, technological advances in stimulation patterns and treatment protocols have not correlated with significant improvements in clinical pain relief outcomes.^47^ As demonstrated in the current animal model study, fMRI-based determination of supraspinal lateralization effects can be used for precise, patient-centric, neuromodulatory interventions. Further, stimulation paradigms for pain control or improvement of motor recovery in SCI patients have often focused on the deafferented cortex.^40^ The current results support the need for an asymmetric focus on either hemisphere for improved neuromodulatory clinical trial designs in SCI patients.

## Supplementary Material

Supplemental data

## Abbreviations Used

ACC: anterior cingulate cortex
BOLD: blood-oxygenation-level–dependent
FCD: functional connectivity density
fMRI: functional magnetic resonance imaging
GE-EPI: gradient-recalled echo planar imaging
i.p.: intraperitoneal
M1/M2: primary and secondary motor cortices
MRI: magnetic resonance imaging
PFC: prefrontal cortex
ROIs: regions of interest
RSFC: resting-state functional connectivity
S1: primary somatosensory cortex
S1-FL: somatosensory forelimb cortex
SCI: spinal cord injury
TE: echo time
TR: repetition time
