# Supplementary material for: Lateralized Supraspinal Functional Connectivity Correlate with Pain and Motor Dysfunction in Rat Hemicontusion Cervical Spinal Cord Injury
Source: Neurotrauma Rep. 2022 Oct 3;3(1):421–32. doi: 10.1089/neur.2022.0040 (PMC9622206; doi:10.1089/neur.2022.0040)
Supplement: Supplemental data [file Suppl_FigS1.docx]

**Figure Caption**

**Supplementary Figure S1.** The 14 anatomical regions of interest (ROIs) considered from the left and right hemispheres separately for the region-based RSFC analysis. 1-PFC-Prefrontal cortex, 2-Ins-Insula, 3-Acc-Anterior cingulate cortex, 4-M1-Primary motor cortex, 5-M2-Secondary motor cortex, 6-S1_FL_-Primary sensory forelimb cortex_,_ 7- S1_HL_-Primary sensory hindlimb cortex_,_ 8-S1_BF_-Primary sensory barrel field cortex_,_ 9-S2-Secondary sensory cortex, 10-Aud-Auditory cortex, 11-Th-Thalamus, HT-12-Hypothalamus, 13-Ag-Amygdala and 14-Hip-Hippocampus. Image orientation is in the radiological convention (image left/right is subject right/left).

**
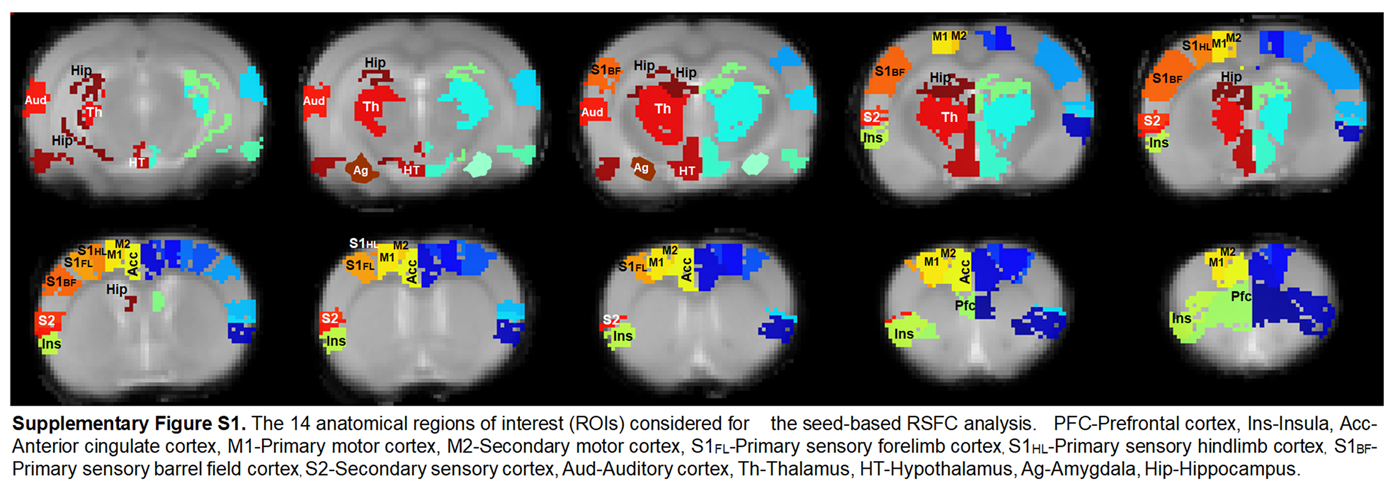
**
